# Contribution of Common *PCSK1* Genetic Variants to Obesity in 8,359 Subjects from Multi-Ethnic American Population

**DOI:** 10.1371/journal.pone.0057857

**Published:** 2013-02-25

**Authors:** Hélène Choquet, Jay Kasberger, Ajna Hamidovic, Eric Jorgenson

**Affiliations:** 1 Ernest Gallo Clinic and Research Center, Emeryville, California, United States of America; 2 Department of Neurology, University of California San Francisco, San Francisco, California, United States of America; 3 Department of Anesthesia, University of California San Francisco, San Francisco, California, United States of America; 4 Department of Preventive Medicine, Northwestern University, Chicago, Illinois, United States of America; Johns Hopkins University, United States of America

## Abstract

Common *PCSK1* variants (notably rs6232 and rs6235) have been shown to be associated with obesity in European, Asian and Mexican populations. To determine whether common *PCSK1* variants contribute to obesity in American population, we conducted association analyses in 8,359 subjects using two multi-ethnic American studies: the Coronary Artery Risk Development in Young Adults (CARDIA) study and the Multi-Ethnic Study of Atherosclerosis (MESA). By evaluating the contribution of rs6232 and rs6235 in each ethnic group, we found that in European-American subjects from CARDIA, only rs6232 was associated with BMI (*P* = 0.006) and obesity (*P* = 0.018) but also increased the obesity incidence during the 20 years of follow-up (HR = 1.53 [1.07–2.19], *P* = 0.019). Alternatively, in African-American subjects from CARDIA, rs6235 was associated with BMI (*P* = 0.028) and obesity (*P* = 0.018). Further, by combining the two case-control ethnic groups from the CARDIA study in a meta-analysis, association between rs6235 and obesity risk remained significant (OR = 1.23 [1.05–1.45], *P* = 9.5×10^−3^). However, neither rs6232 nor rs6235 was associated with BMI or obesity in the MESA study. Interestingly, rs6232 was associated with BMI (*P* = 4.2×10^−3^) and obesity (*P* = 3.4×10^−3^) in the younger European-American group combining samples from the both studies [less than median age (53 years)], but not among the older age group (*P* = 0.756 and *P* = 0.935 for BMI and obesity, respectively). By combining all the case-control ethnic groups from CARDIA and MESA in a meta-analysis, we found no significant association for the both variants and obesity risk. Finally, by exploring the full *PCSK1* locus, we observed that no variant remained significant after correction for multiple testing. These results indicate that common *PCSK1* variants (notably rs6232 and rs6235) contribute modestly to obesity in multi-ethnic American population. Further, these results suggest that the association of rs6232 with obesity may be age-dependent in European-Americans. However, multiple replication studies in multi-ethnic American population are needed to confirm our findings.

## Introduction

Obesity is a common disorder which affects more than 35% of American adults [1] and involves multiple genetic factors [2], [3]. The *PCSK1* (Prohormone Convertase Subtilisin/Kexin type 1) gene is involved in regulation of appetite and consequently in obesity via the biochemical activities of its protein (PC1/3) on key peptides in the leptin-melanocortin pathway [4].

Rare *PCSK1* variants causing total or partial PC1/3 deficiency have been reported to be associated with extreme obesity [5], [6], [7], [8], [9]. Furthermore, common *PCSK1* variants (notably rs6232 and rs6234-rs6235) have been shown to contribute to obesity risk in a study of 13,659 European subjects [10]. Thus, *PCSK1* is considered to play a role in this common disorder. To date, several replication studies have been undertaken in European [11], [12], [13], [14], Asian [15], [16], [17] and Mexican [18] populations. Nevertheless, there is mixed evidence for the association of the rs6232, rs6234 and rs6235 *PCSK1* variants with overweight, obesity and body mass index (BMI).

In Europeans, a first study found a modest association of rs6232 with BMI and obesity in young subjects (age <59 years) from Norfolk, UK [12]. A second study found no significant association between rs6235 and obesity in 3,885 Swedish non-diabetic subjects [11]. A third study reported the association of rs6232 with an increased risk of overweight, and the association of rs6235 with an increased risk of obesity in 3,925 Danish subjects [13]. The initial association of rs6234 with obesity has been recently replicated in 979 Greek subjects [14]. Finally, the GIANT consortium (Genetic Investigation of ANthropometric Traits) detected a modest association between rs6232 and BMI in 32,287 Europeans from 15 cohorts [19]. In Asians, a first study found an association of rs6234 with BMI and overweight in 1,423 Chinese Han men, but not in 1,787 Chinese Han women [15]. A second study reported several common variants in *PCSK1* associated with obesity in 1,094 Chinese individuals [16]. Finally, a recent meta-analysis found no evidence for association between rs6234 or rs6235 and BMI but found an association between rs261967, located near *PCSK1*, and BMI (*P* = 5.13×10^−9^) in 83,048 East Asians [17]. Recently, a study in the Mexican population reported a significant association of rs6232 with obesity in 1,206 children and with class III obesity in 796 adults [18]. These studies provide a mixed view of the association of variants in the *PCSK1* region and obesity-related phenotypes. Further independent studies in different populations could help clarify the association of common variants in *PCSK1* with obesity.

To date, the role of common variants in *PCSK1* in obesity is still unexplored in American population. Our study evaluates the contribution of common variants in *PCSK1* to the risk of obesity in a large multi-ethnic American sample. We assessed the effect of common variants in *PCSK1*, notably the rs6232 and rs6235 polymorphisms on BMI variation and obesity risk in a total of 8,359 Americans from two multi-ethnic studies: the Coronary Artery Risk Development in Young Adults (CARDIA) study and the Multi-Ethnic Study of Atherosclerosis (MESA).

## Materials and Methods

### Study Populations

Written informed consent was obtained from all participants, and study protocols were approved by UCSF Institutional Review Board. Furthermore, this study was approved by CARDIA and MESA Data Access Committees from the National Center for Biotechnology Information Genotypes and Phenotypes Database (NCBI dbGaP, http://www.ncbi.nlm.nih.gov/gap).

### Coronary Artery Risk Development in Young Adults (CARDIA) Study

The CARDIA study was designed to determine the etiology of cardiovascular diseases in 5,115 European and African Americans aged between 18 and 30 years at baseline examination in 1986. The subjects were recruited in four centers: Birmingham, AL; Chicago, IL; Minneapolis, MN; and Oakland, CA [20]. Sample sizes were relatively similar among subgroups of ethnicity, gender, education, and age. Subjects were followed-up every four years during twenty years. Further information is available on the CARDIA website (http://www.cardia.dopm.uab.edu/). After excluding subjects who had missing genotypes at the single nucleotide polymorphisms (SNPs) of interest (rs6232, rs6234 and rs6235) (n = 11) or missing BMI measures, the final sample included 2,448 subjects (1,320 European-Americans and 1,128 African-Americans).

### Multi-Ethnic Study of Atherosclerosis (MESA)

The MESA study was designed to examine the development of atherosclerosis in 6,814 subjects aged between 45 and 84 years at recruitment time and from four ethnicity groups (European-American, African-American, Asian and Hispanic). The subjects were recruited in six centers: Baltimore, MD; Chicago, IL; Forsyth County, NC; Los Angeles, CA; New York, NY; and St Paul, MN. The study population, design and protocols have been previously described [21]. Further information is available on the MESA website (http://www.mesa-nhlbi.org/). After excluding subjects who had missing genotypes at the SNPs of interest (n = 626) or missing BMI measures, the final sample included 5,911 subjects (2,297 European-Americans; 1,645 African-Americans, 672 Asians and 1,297 Hispanics).

### Phenotyping

Standard questionnaires were used to assess demographic information including age, gender and ethnicity for all participants. Weight and height were measured for all participants by trained personnel. BMI was calculated as the weight in kilograms (kg) divided by the square of height in meters (m). Obesity was defined as having a BMI ≥30 kg/m^2^ and lean status was defined as having a BMI <25 kg/m^2^ according to the standard clinical definitions.

### Genotyping

Samples from the CARDIA and MESA studies were genotyped as part of the Candidate Gene Association Resource (CARe) project [22]. For the Phase II of the CARe project, an ITMAT-Broad-CARe (IBC) chip using Illumina iSelect Custom Genotyping BeadChip technology was designed [23]. The IBC chip included 49,320 SNPs from about 2,100 candidate genes selected from the scientific literature, pathway analyses of cardiovascular diseases, and recent results of whole-genome studies. The aim of the Phase II of the CARe project was to analyze the genetic variation within pathways of candidate genes to underpin primary and secondary vascular disease processes. Using the NCBI Build 36 Human reference genome, we defined the *PCSK1* locus (5′ and 3′ intergenic and intragenic regions) as the region between the positions 95,323,531 and 96,023,663 on chromosome 5. At this locus, 31 SNPs were genotyped on the IBC Chip. The three SNPs of interest (rs6232, rs6234 and rs6235) were genotyped on the IBC chip. Since rs6234 and rs6235 were in strong linkage disequilibrium (LD) (r^2^>0.78) in all ethnic groups from the CARDIA and MESA studies and had similar association analysis results, we here only present the results for rs6235. The genotyping call rates of rs6232 and rs6235 were 90% and 99.6% in the MESA and CARDIA cohorts, respectively. Further, the genotype distributions of rs6232 and rs6235 were in Hardy-Weinberg equilibrium in each case-control ethnic groups from the both studies (*P*>0.17 and *P*>0.13, respectively).

### Statistical methods

Using PLINK v1.07 (http://pngu.mgh.harvard.edu/purcell/plink/) [24], we evaluated the associations of the above-mentioned SNPs with BMI variation and obesity risk by linear and logistic regression under an additive model, and adjusting for age, gender and center. To correct for population stratification, we also included the first ten principal components as covariates in all the genetic association analyses. To calculate the ten principal components, we used PCA as implemented in EIGENSTRAT [25] on the cleaned CARe IBC genotype data, without additional filtering. Together with the CARe samples, we analyzed genotype data from the HapMap populations (CEU, YRI, CHB+JPT), all genotyped on IBC. The HapMap populations were used as reference populations. Default parameters in the SMARTPCA program were also used [26]. Non-normality of the BMI quantitative trait was corrected by Box-Cox transformation [27], [28] and scaled to a mean of 0 and a standard deviation of 1 using the R v2.12.1 software (http://www.r-project.org) (The R Foundation for Statistical Computing, Vienna, Austria, 2011). Survival curves were modeled and analyzed using the Cox proportional hazards regression model using R v2.12.1 software. Association analyses at the *PCSK1* locus were corrected for multiple testing (Bonferroni correction) by using the “adjust” option in PLINK. The statistical power of the different tests was determined using the QUANTO software (http://hydra.usc.edu/gxe/) and the linkage disequilibrium between SNPs in *PCSK1* was estimated using PLINK and Haploview v4.2 (http://www.broad.mit.edu/mpg/haploview). For the meta-analysis of obesity, fixed effects or random effects summary estimates were calculated for an additive model using R package “meta”. Heterogeneity index, *I^2^* (0–100%) was assessed among studies and *P*-value for Cochrane's Q statistic was estimated. To evaluate the percentage of genetic variability tagged by the SNPs genotyped at the *PCSK1* locus and intergenic regions (chr5:95,323,531–96,023,663 using NCBI Build 36), we used Haploview v4.2 software. We selected genotype tagging SNPs (tag SNPs) to capture known variation with MAF>0.05 and with an r^2^>0.8 in HapMap populations.

## Results

### Population-based studies

Clinical characteristics of the studied populations are described in Table 1. Because the mean age at last exam for the CARDIA subjects was the closest to the mean age at baseline for the MESA subjects, we decided to use the CARDIA data at last exam to study associations. Furthermore, the proportion of obese subjects in the CARDIA study was the largest at the last exam. Consequently, the association test using the data at the last exam is the most statistically powerful to detect the initial effects previously reported [10].

**Table 1:** Description of the populations used for association analyses.

Population	Ethnic Group	N total (female)	Status	Age[years] (mean ± SD)	BMI [kg/m^2^] (mean ± SD)
CARDIA *at last exam*	European-American	1,320 (698)	—	40.75±3.36	27.12±5.89
		263 (146)	Case	40.89±3.43	36.42±5.14
		547 (353)	Control	40.62±3.38	22.28±1.84
	African-American	1,128 (669)	—	39.53±3.83	30.65±7.49
		453 (313)	Case	39.86±3.96	37.84±6.26
		251 (135)	Control	38.97±3.87	22.40±1.98
MESA *at baseline*	European-American	2,297 (1,098)	—	62.65±10.25	27.74±5.06
		631 (311)	Case	61.07±9.61	34.19±3.82
		738 (258)	Control	62.71±10.71	22.66±1.76
	African-American	1,645 (745)	—	62.15±10.09	30.15±5.88
		744 (267)	Case	61.02±9.77	35.20±4.58
		293 (156)	Control	63.77±10.66	22.75±1.82
	Hispanic	1,297 (621)	—	61.32±10.31	29.43±5.11
		505 (201)	Case	60.64±9.82	34.40±4.20
		219 (106)	Control	62.09±11.14	23.06±1.51
	Asian	672 (336)	—	62.59±10.44	23.92±3.28
		27 (13)	Case	62.07±8.84	31.69±2.58
		439 (219)	Control	62.92±10.48	22.05±1.98

The rs6232 was largely monomorphic (MAF ≈ 1%) in African-Americans from CARDIA and MESA cohorts and in Asians from MESA cohort. Thus, rs6232 was excluded from the analysis for both of these ethnic groups. Furthermore, the number of obese subjects for MESA Asians was limited (N = 27) and did not provide a sufficient sample size for the obesity case-control analysis for the rs6235 variant.

### CARDIA follow-up study

**Figure 1:**
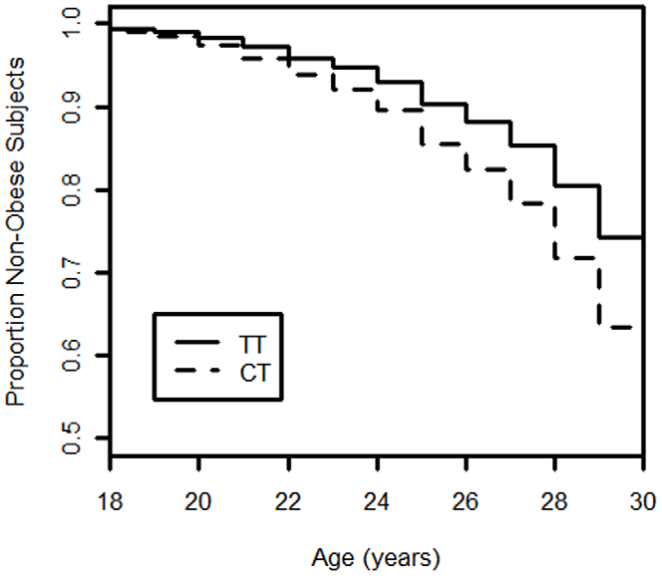
Obesity incidence, by age at baseline in European-Americans from CARDIA according to the rs6232 genotype. The proportion with obesity was calculated within each genotypic class to assess the impact of the genotype on obesity incidence. An increase incidence of obesity was confirmed by Cox survival analysis (adjusted for gender and center) among TT and CT carriers during the 20 years of follow-up (hazard ratio 1.53 [1.07–2.19], *P* = 0.019).

To determine whether rs6232 and rs6235 contribute to obesity in the CARDIA study, we first assessed the effect of these two SNPs on BMI variation and obesity risk in 1,320 European-Americans from CARDIA at the last exam. We found that BMI was higher in subjects carrying the minor rs6232 at-risk allele (*P* = 0.006). We also detected an increased risk of obesity only for the rs6232 variant (OR = 1.71 [1.09–2.67], *P* = 0.018). We then assessed the effect of rs6235 in 1,128 African-Americans from CARDIA and we found that BMI was higher in subjects carrying the minor rs6235 at-risk allele (*P* = 0.028). We also observed that the same rs6235 allele was associated with a significantly increased risk of obesity (OR = 1.47 [1.07–2.03], *P* = 0.018). Results are reported in Table 2. By combining the two case-control ethnic groups from the CARDIA study in a meta-analysis, association between rs6235 and obesity risk remained significant (OR = 1.23 [1.05–1.45], *P* = 9.5×10^−3^). As we did not detect significant heterogeneity between the two ethnic groups (I*^2^* = 57.2%, *P* = 0.13), we reported values for fixed effects model for this meta-analysis. Additionally, we tested the effect of the two above-mentioned *PCSK1* SNPs on the incidence of obesity among each ethnic group using Cox survival analysis, adjusted for gender and center. Individuals who were non-obese at baseline were then reanalyzed after 20 years of follow-up, and we compared those who remained non-obese at the end of the study with the incident obese participants. In CARDIA European-Americans, an increase in obesity incidence was found for the rs6232 at-risk allele during the 20 years of follow-up (hazard ratio = 1.53 [1.07–2.19], *P* = 0.019) (Figure 1), but not for the rs6235 at-risk allele (HR = 1.04 [0.85–1.27], *P* = 0.730). In CARDIA African-Americans, only the rs6235 SNP was analyzed and we did not observe an effect of this variant on the incidence of obesity during the 20 years of follow-up (HR = 1.07 [0.88–1.29], *P* = 0.520) (Table 3). These findings indicate that rs6232 and rs6235 *PCSK1* polymorphisms contribute modestly to obesity in the CARDIA study. Furthermore, these results show that association of rs6232 and rs6235 variants with obesity may be ethnicity-specific.

**Table 2:** Genotype distributions of the two *PCSK1* variants in the CARDIA study.

			Genotype distributions				Additive	
rs6232			TT	TC	CC	MAF (%)	β/OR [95%C.I.]	*P*
European-Americans	BMI	n	1,172	148	0	5.6	—	—
		mean ± s.d.	26.98±5.81	28.39±6.35	—	—	0.24 [0.07–0.41]	0.006
	Obesity	Controls	494	53	0	4.8	—	—
		Cases	222	41	0	7.8	1.71 [1.09–2.67]	0.018
rs6235			CC	CG	GG	MAF (%)	β/OR [95%C.I.]	*P*
European-Americans	BMI	n	693	514	113	28.0	—	—
		mean±s.d.	26.94±5.73	27.29±5.95	27.70±6.48	—	0.05 [−0.03–0.14]	0.191
	Obesity	Controls	288	217	42	27.5	—	—
		Cases	130	108	25	30.0	1.13 [0.90–1.43]	0.292
African-Americans	BMI	n	786	315	27	16.3	—	—
		mean±s.d.	30.48±7.63	31.03±7.27	32.34±5.92	—	0.13 [0.01–0.24]	0.028
	Obesity	Controls	183	66	2	13.9	—	—
		Cases	302	136	15	18.3	1.47 [1.07–2.03]	0.018
Meta-Analysis	Obesity	—	—	—	—	—	1.23 [1.05–1.45]	9.5×10^−3^

*P*-values are adjusted for age, gender center and the first 10 principal components.

**Table 3:** *PCSK1* polymorphisms association with obesity incidence during a 20-year follow-up of the CARDIA study.

		Genotype distributions			Additive	
rs6232		TT	TC	CC	HR [95%C.I.]	*P*
European-Americans	Controls	902	100	0	—	—
	Cases	189	36	0	1.53 [1.07–2.19]	0.019
rs6235		CC	CG	GG	HR [95%C.I.]	*P*
European-Americans	Controls	533	386	83	—	—
	Cases	116	91	18	1.04 [0.85–1.27]	0.730
African-Americans	Controls	429	158	11	—	—
	Cases	223	103	11	1.07 [0.88–1.29]	0.520

Controls: participants followed 20 years and remaining non-obese (BMI<30 kg/m^2^) over the 20 years.

Cases: incident cases of obesity over the 20 years.

HR: hazard ratio.

*P*-values are adjusted for gender and center.

### MESA cohort

To determine whether rs6232 and rs6235 *PCSK1* polymorphisms contribute to obesity in the MESA cohort, we performed association analyses with BMI variation and obesity risk in each ethnic group (European-Americans, African-Americans, Asians and Hispanics) of the MESA cohort. We found that neither of these two above-mentioned SNPs was associated with BMI variation or obesity risk in any of the ethnic groups. By combining all case-control ethnic groups from MESA study in a meta-analysis (N = 2,093 and 3,596 for the rs6232 and rs6235 variants respectively) we did not observe significant association of the both SNPs with obesity risk (OR = 1.05 [0.79–1.40], *P* = 0.72; OR = 0.99 [0.89–1.10], *P* = 0.83; for rs6232 and rs6235, respectively). As we did not detect heterogeneity between the ethnic groups (I*^2^* = 0%, *P* = 0.34 and I*^2^* = 11.8%, *P*  = 0.32 for rs6232 and rs6235, respectively), we reported values for fixed effects model. Results are presented in Table 4.

**Table 4:** Genotype distributions of the two *PCSK1* variants in the MESA study.

			Genotype distributions				Additive	
rs6232			TT	TC	CC	MAF (%)	β/OR [95%C.I.]	*P*
European-Americans	BMI	n	2,107	187	3	4.2	—	—
		mean±s.d.	27.74±5.09	27.81±4.80	28.69±4.90	—	0.02 [−0.12–0.17]	0.733
	Obesity	Controls	676	61	1	4.3	—	—
		Cases	571	59	1	4.8	1.12 [0.77–1.63]	0.543
Hispanic-Americans	BMI	n	1,242	55	0	2.1	—	—
		mean±s.d.	29.51±5.17	28.51±4.99	—	—	−0.13 [−0.41–0.14]	0.330
	Obesity	Controls	209	10	0	2.3	—	—
		Cases	488	17	0	1.7	0.77 [0.34–1.77]	0.542
Meta-Analysis	Obesity	—	—	—	—	—	1.05 [0.79–1.40]	0.720
rs6235			CC	CG	GG	MAF (%)	β/OR [95%C.I.]	*P*
European-Americans	BMI	n	1,246	892	159	26.3	—	—
		mean±s.d.	27.75±5.09	27.63±4.98	28.40±5.39	—	0.02 [−0.05–0.08]	0.567
	Obesity	Controls	389	304	45	26.7	—	—
		Cases	335	241	55	27.8	1.02 [0.86–1.22]	0.785
African-Americans	BMI	n	1,140	422	48	16.1	—	—
		mean±s.d.	30.27±5.82	30.06±5.98	29.68±6.92	—	−0.04 [−0.14–0.05]	0.366
	Obesity	Controls	197	81	12	18.1	—	—
		Cases	529	192	17	15.3	0.86 [0.66–1.12]	0.276
Hispanics	BMI	n	822	425	50	20.2	—	—
		mean±s.d.	29.30±4.93	29.78±5.65	29.58±4.47	—	0.08 [−0.02–0.17]	0.122
	Obesity	Controls	144	69	6	18.5	—	—
		Cases	323	163	19	19.9	1.08 [0.80–1.47]	0.611
Asians	BMI	n	309	289	74	32.0	—	—
		mean±s.d.	23.95±3.50	23.81±3.07	24.22±3.18	—	0.02 [−0.09–0.14]	0.700
	Obesity	Controls	201	195	43	32.0	—	—
		Cases	15	10	2	26.0	NA	NA
Meta-Analysis	Obesity	—	—	—	—	—	0.99 [0.89–1.10]	0.830

*P*-values are adjusted for age, gender, center and the first 10 principal components.

NA: not available.

### Age-Dependent Effect of rs6232 on Obesity

To assess a potential age-dependent effect of rs6232 on obesity as previously reported in Europeans from UK [12], we performed association test with age stratification. We first analyzed the association of rs6232 with BMI or obesity in the younger and older individuals separately, stratified by the median age of each ethnic group (41 years for CARDIA European-Americans, 63 and 61 years for MESA European-Americans and Hispanics, respectively). Interestingly, rs6232 was modestly associated with BMI in the younger age group (β = 1.93 [0.35–3.52], *P* = 0.017), but not among the older individuals (β = 1.04 [−0.25–2.34], *P* = 0.116) in European-Americans from CARDIA. Consistently, we found a borderline association between rs6232 and obesity in the younger age group (OR = 1.93 [0.99–3.78], *P* = 0.054), but not among the older individuals (OR = 1.44 [0.78–2.66], *P* = 0.239). However, no significant difference was observed between the younger and the older age groups for association between rs6232 and BMI/obesity in European-Americans and Hispanics from MESA. We then combined samples of European-Americans from both cohorts, and we found that rs6232 was significantly associated with BMI in the younger age group (less than 53 years) (β = 1.22 [0.38–2.05], *P* = 4.2×10^−3^), but not among the older individuals (β = −0.13 [−0.99–0.72], *P* = 0.756). Consistently, a significant association between rs6232 and obesity was detected in the younger age group (OR = 1.75 [1.20–2.55], *P* = 3.4×10^−3^), but not among the older individuals (OR = 0.98 [0.64–1.51], *P* = 0.935). As a note, all subjects in the older age group were from MESA cohort. Identical analyses were performed for rs6235 but no effect age-dependent on BMI/obesity was detected for this variant. Our results show an effect of rs6232 on obesity risk only in younger European-Americans.

### Meta-analysis

To increase statistical power, we first tested the effect of rs6232 and rs6235 on obesity risk by combining all the case-control ethnic groups from CARDIA and MESA in a meta-analysis (N = 2,903 and 5,110 for rs6232 and rs6235, respectively). Because of the heterogeneity between the groups (I*^2^* = 60%, *P* = 0.08 and I*^2^* = 59.2%, *P* = 0.04 for rs6232 and rs6235, respectively), we used a random effects model. No significant association between the two SNPs and obesity risk was found under an additive model (OR = 1.22 [0.82–1.80], *P* = 0.32; OR = 1.08 [0.93–1.24], *P* = 0.31 for rs6232 and rs6235, respectively), despite acceptable statistical power (62.0 and 98.8%) to detect the effects with obesity previously reported, at a significance of 5% [10]. We then combined subgroups of the same ethnicity from CARDIA and MESA in a meta-analysis. No significant association between the two SNPs and obesity risk was found (OR = 1.37 [0.90–2.07], *P* = 0.14; OR = 1.05 [0.94–1.18], *P* = 0.35 for rs6232 and rs6235, respectively) in 2,179 European-American subjects. Further, no significant association between rs6232 and obesity risk was found (OR = 1.12 [0.66–1.89], *P* = 0.68) in 1,741 African-American subjects. Our results confirm that these two variants of *PCSK1* are not essential contributors to obesity risk in a large multi-ethnic American population.

### Full *PCSK1* locus exploration

Finally, we asked whether other common *PCSK1* variants contribute to obesity in multi-ethnic American population. To answer this question, we explored the entire *PCSK1* locus by evaluating the effects of the *PCSK1* SNPs available for the GWAS data set described above, on obesity risk. At the entire *PCSK1* locus, 31 SNPs were genotyped on the IBC Chip. These SNPs represented a minimum of 37% of the genetic variability at the entire *PCSK1* locus based on HapMap populations. For each polymorphism, we performed association analyses with BMI and obesity in each ethnic group described above. The list of *PCSK1* SNPs genotyped for the GWA studies and the results of association analyses with obesity are presented in Table S1. We found no association between *PCSK1* variant and BMI or obesity that remained significant after correction for multiple testing (*P*≤1.61×10^−3^), either in CARDIA or in MESA ethnic groups.

## Discussion

The results of this study indicate that common *PCSK1* variants, notably the rs6232 and rs6235 polymorphisms contribute modestly to obesity in multi-ethnic American population. First, rs6232 was associated with BMI variation and obesity risk in European-American subjects from the CARDIA study. Further, despite a statistical power of 63.1%, at a significance of 5%, we found that rs6235 was modestly associated with BMI variation and obesity risk in CARDIA study. However, neither rs6232 nor rs6235 was associated with BMI or obesity in MESA study. Moreover, by combining all the case-control ethnic groups from CARDIA and MESA in a meta-analysis, we found no significant association for the both variants and obesity risk. Finally, no significant association with BMI variation or obesity risk was observed for any of the 31 SNPs genotyped at the entire *PCSK1* locus. Thus, common variation at *PCSK1* gene is not an essential contributor to the risk of obesity in multi-ethnic American population.

Consistently with previous findings showing an age-dependent effect of rs6232 on obesity [12], we found that rs6232 was associated with BMI and obesity only in the younger European-American group (less than 53 years) combining samples from CARDIA and MESA. As we indicated, all combined European-American subjects in the older age group were from MESA study. Further, the mean age of subjects in the MESA study was 62 years. Thus, the age-dependent effect of rs6232 on obesity may in part explain the lack of association in the elder MESA study for this variant.

Our results extend previous findings showing that the initial association of rs6232 and rs6235 with obesity have subsequently been reproduced in some but not all European [11], [12], [13], [14], Asian [15], [16], [17] or Mexican [18] studies. Indeed, there are a variety of possible reasons to explain the lack of replication between studies, notably between CARDIA and MESA studies. These include differences in ascertainment or study design, population substructure, genotype call rate, and heterogeneity between the studies due to unknown genetic, lifestyle, or environmental factors [29], [30]. Thus, heterogeneity may explain the lack of replication in MESA study despite a larger sample size.

Another interesting finding of the present study is that for the first time, we report the contribution of common *PCSK1* variants in multi-ethnic American population. Interestingly, we found that the minor allele frequencies of rs6232 and rs6235 are relatively different among ethnic groups (MAF_rs6232_≈4% in European-Americans; MAF_rs6232_≈1% in African-Americans; MAF_rs6232_ = 2.1% in Hispanics and MAF_rs6232_≈1% in Asians; MAF_rs6235_≈26% in European-Americans; MAF_rs6235_≈16% in African-Americans; MAF_rs6235_ = 20% in Hispanics and MAF_rs6235_ = 32% in Asians). Similarly, a recent study reported significant differences between minor allele frequencies of rs6232 and rs6234 in Aboriginal and European populations of Northern Ontario [31]. Moreover, our findings are consistent with the previously reported frequencies for rs6232 and rs6235 in Asian subjects [15], [16]. Indeed, rs6232 is uncommon in Asian populations. Our finding of the lack of replication with BMI for rs6235 in Asians from MESA is consistent with the previous studies in Asian populations [16], [17]. Indeed, none of these studies has reported a significant association of rs6235 with BMI or obesity [16], [17]. The rs6232 and rs6235 *PCSK1* variants may have variable effects according to ethnicity groups. Thus, additional studies in diverse populations are warranted.

In conclusion, our study has provided first insights of the modest contribution of common *PCSK1* variation to obesity in multi-ethnic American population. Our results suggest that the association of obesity with rs6232 may be age-dependent in European-Americans. Further replication studies are essential to confirm the association between genetic variation in *PCSK1* and obesity in multi-ethnic American population.

## Supporting Information

Table S1 ***PCSK1*** **polymorphisms genotyped on the IBC chip and association results with obesity**.
(DOCX)
