# Supplementary material for: Contribution of Common PCSK1 Genetic Variants to Obesity in 8,359 Subjects from Multi-Ethnic American Population
Source: PLoS One. 2013 Feb 25;8(2):e57857. doi: 10.1371/journal.pone.0057857 (PMC3581482; doi:10.1371/journal.pone.0057857)
Supplement: Table S1 — PCSK1 polymorphisms genotyped on the IBC chip and association results with obesity. (DOCX) [file pone.0057857.s001.docx]

**Table S1.** ***PCSK1* polymorphisms genotyped on the IBC chip and association results with obesity**

| **NCBI dbSNP** | **Position^a^** | **CARDIA** | | **MESA** | | | |
| --- | --- | --- | --- | --- | --- | --- | --- |
|  |  | **Europeans** | **Africans** | **Europeans** | **Africans** | **Hispanics** | **Asians** |
| **rs10515236** | **95752619** | NA | 0.973 | NA | 0.239 | NA | NA |
| **rs10515237** | **95777305** | 0.240 | 0.301 | 0.240 | 0.109 | 0.227 | 0.603 |
| **rs11741888** | **95756217** | 0.179 | 0.622 | 0.211 | 0.342 | 0.622 | 0.879 |
| **rs13159579** | **95790446** | 0.188 | NA | 0.203 | NA | NA | 0.269 |
| **rs13361555** | **95765297** | NA | 0.386 | NA | 0.296 | 0.787 | NA |
| **rs1391808** | **95763072** | NA | 0.052 | NA | 0.644 | 0.477 | NA |
| **rs1498928** | **95776413** | 0.572 | 0.772 | 0.401 | 0.206 | 0.913 | 0.569 |
| **rs155438** | **95799387** | 0.993 | 0.885 | 0.678 | 0.070 | 0.060 | 0.885 |
| **rs155968** | **95778316** | 0.917 | 0.094 | 0.383 | 0.059 | 0.110 | 0.785 |
| **rs155979** | **95795654** | 0.057 | 0.614 | 0.150 | 0.149 | 0.402 | 0.931 |
| **rs155982** | **95797051** | NA | NA | NA | NA | NA | 0.931 |
| **rs155994** | **95786744** | 0.194 | 0.022 | 0.042 | 0.770 | 0.678 | 0.857 |
| **rs156016** | **95768370** | 0.835 | 0.903 | 0.449 | 0.074 | 0.194 | 0.715 |
| **rs17085675** | **95753420** | 0.292 | 0.014 | 0.634 | 0.015 | 0.685 | 0.382 |
| **rs2242385** | **95794861** | NA | NA | NA | NA | NA | 0.219 |
| **rs271920** | **95758742** | 0.801 | 0.164 | 0.265 | 0.903 | 0.655 | 0.885 |
| **rs271923** | **95763118** | 0.163 | 0.579 | 0.147 | 0.441 | 0.581 | 0.633 |
| **rs271926** | **95763430** | 0.901 | 0.464 | 0.269 | 0.249 | 0.645 | 0.885 |
| **rs271927** | **95767557** | 0.181 | 0.093 | 0.072 | 0.644 | 0.568 | 0.946 |
| **rs3762983** | **95795851** | 0.536 | 0.310 | 0.049 | 0.555 | 0.345 | 0.720 |
| **rs3792744** | **95768069** | 0.200 | 0.801 | 0.108 | 0.541 | 0.756 | 0.756 |
| **rs3792747** | **95793676** | 0.139 | 0.948 | 0.255 | 0.620 | 0.808 | 0.524 |
| **rs3811945** | **95785316** | NA | NA | NA | NA | NA | 0.945 |
| **rs3811948** | **95786981** | NA | NA | NA | NA | NA | 0.998 |
| **rs3811952** | **95792138** | NA | NA | NA | NA | 0.699 | 0.409 |
| **rs436321** | **95780239** | 0.208 | 0.006 | 0.051 | 0.534 | 0.608 | 0.838 |
| **rs4869134** | **95798227** | 0.222 | 0.409 | 0.193 | 0.294 | 0.148 | 0.476 |
| **rs6232** | **95777541** | 0.019 | NA | 0.543 | NA | 0.542 | NA |
| **rs6233** | **95758868** | 0.834 | 0.140 | 0.265 | 0.807 | 0.621 | 0.885 |
| **rs6234** | **95754730** | 0.292 | 0.040 | 0.758 | 0.122 | 0.697 | 0.374 |
| **rs6235** | **95754654** | 0.292 | 0.018 | 0.785 | 0.276 | 0.611 | 0.374 |

**^a^**Chromosome 5q, NCBI Build 36

Data are *P*-values adjusted for age, gender, center and the first 10 principal components

NA: not available (MAF≤1% or low genotype quality score)
